# Study of pH and Thermodynamic Parameters via Circular Dichroism Spectroscopy of a Recombinant Human Lactoferrin

**DOI:** 10.3390/molecules29020491

**Published:** 2024-01-19

**Authors:** Beatriz L. Álvarez-Mayorga, Sergio Romero-Gómez, Jorge L. Rosado, Janet Ocampo-Hernández, J. Gómez-Guzmán, Luis Ortiz-Frade

**Affiliations:** 1 Facultad de Química, Universidad Autónoma de Querétaro, Queretaro 76010, Mexico; sergio.romero@uaq.edu.mx; 2 Departamento de Nutrición Humana, Facultad de Ciencias Naturales, Universidad Autónoma de Querétaro, Queretaro 76230, Mexico; 3 Departamento de Electroquímica, Centro de Investigación y Desarrollo Tecnológico en Electroquímica S.C. Parque Tecnológico Querétaro, Sanfandila, Queretaro 76703, Mexico; jocampo@cideteq.mx (J.O.-H.); jesusgomezguzman@hotmail.com (J.G.-G.); lortiz@cideteq.mx (L.O.-F.)

**Keywords:** circular dichroism, protein denaturalization, recombinant proteins, human lactoferrin, *Komagataella phaffii*

## Abstract

The production of human recombinant proteins to be used for therapeutic or nutritional purposes must focus on obtaining a molecule that is as close as possible to the native human protein. This biotechnological tool has been documented in various studies published in recent decades, with lactoferrin being one of those that has generated the most interest, being a promising option for recombinant technology. However, stability studies including thermodynamic parameters have not been reported for recombinant lactoferrin (Lf). The objective of this work was to obtain the human recombinant protein using the yeast *Komagataella phaffii* to study structural changes modifying pH and temperature using circular dichroism spectroscopy (CD). Thermodynamic parameters such as ΔH, ΔS and Tm were calculated and compared with commercial human lactoferrin. We propose the potential use of CD and thermodynamic parameters as a criterion in the production of recombinant proteins to be used in the production of specialized recombinant proteins.

## 1. Introduction

Lactoferrin is a non-heme iron-binding glycoprotein, belonging to the transferrin family, which is secreted in the milk of most mammals and is also present in the secretions of mucosal surfaces. This multifunctional protein is an essential component of the host defense innate mechanisms. Lactoferrin sequesters iron from the medium, thus avoiding the growth of microorganisms, and it can also interact with the bacterial membrane, causing its destabilization [1].

Independently of iron-binding capability, lactoferrin interacts with microbial, viral and cell surfaces, thus inhibiting its microbial and viral adhesion and entry into host cells. Lactoferrin can be considered not only a primary defense factor against mucosal infections, but also a polyvalent regulator which interacts in viral infectious processes [2]. Lactoferrins also have broad activity against capsulated and naked viruses that cause diseases in both humans and animals. The mechanisms of the antiviral activities, lactoferricins, and lactoferrin derived peptides include the prevention of virus-induced apoptosis of cells and the prevention of virus entry into cells by binding to virus envelope proteins or to the virus receptors on the cells or by influencing other intracellular virus mechanisms [3]. Its antiviral activity, lies in the early phase of infection, thus preventing entry of the virus into the host cell. This activity is exerted by binding to heparan sulphate glycosaminoglycan cell receptors or viral particles or both. Despite the antiviral effect of lactoferrin, widely demonstrated in in vitro studies, few clinical trials have been carried out, and the related mechanism of action is still under debate. The nuclear localization of lactoferrin in different epithelial human cells suggests that lactoferrin exerts its antiviral effect not only in the early phase of virus–cell surface interaction, but also intracellularly. The capability of lactoferrin to exert potent antiviral activity through its binding to host cells and/or viral particles, and its nuclear localization, strengthen the idea that lactoferrin is an important brick in the mucosal wall, effective against viral attacks and could be usefully applied as a novel strategy for the treatment of viral infections [2]. Inhibitory effects of lactoferrin towards the influenza A viruses H1N1, H3N2, H5N1, hepatitis C virus, hepatitis B virus, herpes simplex virus, human cytomegalovirus, human immunodeficiency virus, coronavirus, adenovirus, human papilloma virus, rotaviruses, echoviruses, have been reported [3].

Lactoferrin shows different mechanisms for its antiviral activities against the influenza A viruses, bovine lactoferrin prevents virus spread, potentially through the prevention of caspase 3 activity, this enzyme is a cysteinyl protease that has one of the crucial roles in the regulation of apoptosis; furthermore, it has been shown that lactoferrin interacts with influenza A virus hemagglutinin and prevents infection by different H1 and H3 viral subtypes. It has been showed that both bovine and human lactoferrin bound equally well to two glycoproteins in the surface envelope of hepatitis C virus E1 y E2. The mechanism of herpes simplex virus entry into host cells is mediated by virus attachment to glycosaminoglycans as mostly heparan sulfate, in cell surface receptor proteoglycans, via the viral glycoproteins gB and gC, studies indicated that human lacoferrin can bind to heparan sulfate and/or chondroitin sulfate glycosaminoglycans in the cell membranes to occupy the initial places of virus binding. It appears that a globular structure and a net negative charge are both required for anti-human immunodeficiency virus-1 activity, lactoferrins interfere with virus replication, probably at the level of virus-cell fusion and/or binding to thus prevent virus entry into host cells. Antiviral activities of lactoferrins against non-enveloped viruses were first reported for rotavirus, bovine lactoferrin prevented rotavirus hemagglutination and viral binding to susceptible cells by binding to the viral particles [3].

Recently, it has become even more relevant, given that there is evidence that it can also bind to some receptors used by coronaviruses and block their entry, in such a way that lactoferrin could prevent acute respiratory syndrome caused by the SARS-CoV-2 virus, preventing it from adhering to host cells [4]. Furthermore, in a study by Hou et al., they found evidence that lactoferrin can induce osteoblast proliferation while inhibiting apoptosis [5]. Among the trophic factors in human milk, Lf is an important bioactive molecule regulating small intestinal growth (proliferation and differentiation), mediated by a tightly regulated Lf receptor on the plasma membrane of both epithelial cells and crypt cells [6]. The application of lactoferrin as a biologically active protein is expanding. The use of lactoferrin in the food industry should be considered promising for the provision of food products with an iron-binding component, the enhancement of their immunomodulating characteristics and antioxidant action, as well as increasing product shelf-life [7]. According to the characteristics of lactoferrin, a variety of separation methods have been used for the purification of lactoferrin, including salt-out, organic reagent precipitation and chromatographic methods [8,9,10,11,12]. There are some other separation methods, such as membrane technologies, magnetic nanoparticles, etc., and there are even studies where combined methods are used. However, they all present great advantages and disadvantages. 

On the other hand, if a foreign recombinant protein is introduced into the human body, it may trigger an immune response. It is not clear whether the activity of bovine bLF is the same as that of human hLF for all proposed functions. In contrast, there is evidence that bLF is quite different in several aspects compared to hLF, and it has been observed mechanistically that bLF does not bind to the same site on enterocytes in an infant’s small intestine [13]. The production of lactoferrin by genetic engineering technology has become a research hotspot and development trend to produce lactoferrin and avoid its side effects [14].

The production of recombinant proteins in eukaryotic systems is well documented, specifically using yeasts such as *Pichia pastoris*, now renamed *Komagataella phaffi*; the yeast species *Pichia* was transferred to the monotypic genus *Komagataella* and was represented by two species, *K. pastoris* and *K. phaffi* [15]. In this yeast, high levels of expression of proteins such as human lactoferrin have been reported with different expression vectors, such as pPIC9K, among others [16,17]. Obtaining high yields of a recombinant protein has always been the most important challenge. In the last decade, various investigations have been carried out to obtain lactoferrin through genetic engineering. These systems have included bacterial, fungal, plant and animal cells [8]. The sequence of lactoferrin derived from humans, bovines, sheep, goats, pigs, horses, mice, buffalo and camels has been resolved [14,18]. Lactoferrins from mammals have similar amino acid sequences. For example, human and bovine lactoferrin have 691 and 696 amino acids, respectively, with ~70% sequence identity [19].

For this family of recombinant proteins, there are few studies on simple structural characterization; studies are reported of equine recombinant proteins produced in yeast [20]; human recombinant proteins produced in transgenic animals [21]; and native goat, bovine or even human proteins [22]. 

Circular dichroism (CD) of a protein can give structural information on proteins [20,21]. The near-UV CD of a protein generally reflects its secondary structure content and provides a valuable fingerprint of the tertiary structure of that protein, which can be used to compare, for example, mutant and native protein forms [9,20]. It involves a simple way to prepare the samples and requires low-cost equipment, which are some of its advantages in comparison with X-rat diffraction methods [22].

In the secondary structure of a protein, the peptide bond arrangement is highly ordered, and the directionality of the peptide bond arrangement also determines the transition and splitting of the energy level of the peptide bond. Therefore, different secondary structural features of proteins result in different CD band positions and absorption intensities. According to the far-ultraviolet CD of the detected protein, the information of the protein’s secondary structure can be reflected [22,23].

Genetic constructs containing the human lactoferrin (hLF) gene were created within a joint program of Russian and Belorussian scientists. Using these constructs, transgenic mice were bred (the maximum hLF concentration in their milk was 160 g/L), and transgenic goats were also generated (up to 10 g/L hLF in their milk). Experimental goat herds that produced hLF in their milk were also bred, and the recombinant hLF was found to be identical to the natural protein in its physical and chemical properties. These properties included, among other parameters, circular dichroism spectroscopy studies [24].

Paramasivam et al. compared the circular dichroism pattern of recombinant and native equine lactoferrins, and the calculated values for the content of the secondary structural elements of the recombinant protein matched the values obtained for the native protein. All these results confirmed that the expressed protein was properly folded and functional [20]. It is also interesting to evaluate whether similar data are found with human lactoferrin. However, stability studies including thermodynamic parameters have not been reported for recombinant lactoferrin (Lf).

The aforementioned encouraged us in this work to obtain the human recombinant protein using the yeast *Komagataella phaffii* in order to a study structural changes modifying pH and temperature using circular dichroism spectroscopy (CD). Thermodynamic parameters such as ΔH and ΔS were calculated and compared with commercial human lactoferrin (hLF).

## 2. Results and Discussion

### 2.1. Production of Recombinant Proteins

The proportion of the culture medium with respect to the volume of the flask and the agitation must be considered so that the aeration of the culture medium allows for the optimal development of the yeast and therefore the production of the protein of interest [25,26].

#### 2.1.1. Protein Expression

*Komagaetella phaffii*, a methylotrophic yeast, is particularly suited to foreign protein expression for a number of reasons, including ease of genetic manipulation, e.g., gene targeting; high-frequency DNA transformation; cloning via functional complementation; high levels of protein expression at the intra- or extracellular level; and the ability to perform higher eukaryotic protein modifications, such as glycosylation, disulfide bond formation and proteolytic processing [26]. 

To ensure that the *K. phaffii* X-33 yeast strains of interest incorporated the hLF recombinant gene into their genome, the region encompassing the GAP promoter and the AOX region of the pGZaLM plasmid were amplified by endpoint PCR. Genomic DNA was extracted using a PureLink^®^ Genomic DNA Kit (Thermo Fisher Scientific, Waltham, MA, USA). In the electrophoretic analysis (Figure S1) it was observed that the fragment amplified by PCR corresponds to the theoretical size of 2500 bp expected for the optimized hLF nucleotide sequence.

#### 2.1.2. Obtaining Purified hLF Recombinant Protein

The extracts obtained were concentrated in AMICON^TM^ Ultracel 10 K tubes (Merck, Darmstadt, Germany). The procedure was performed twice and the total protein in both the crude extract and the concentrate was quantified.

The production of lactoferrin had a low yield in the culture medium (0.186 mg/mL). With twice the concentration added to an AMICON^TM^ tube, a concentration of 0.673 mg/mL was obtained (Figure S2). The data found in the literature include recombinant protein yields using the Pichia expression system of 40 mg/L of equine lactoferrin and 12 mg/L of porcine lactoferrin, both obtained in a bioreactor [26]; however, there are no data for recombinant human lactoferrin. 

Figure S3 shows the secretion of the human recombinant protein lactoferrin, which has a relative mobility similar to that of the bovine standard, due to the fact that recombinant hLF, being a fusion protein, also has the myc epitope and the polyhistidine tail that allow for identification with antibodies and specific purification, respectively. 

### 2.2. Circular Dichroism

#### 2.2.1. Effect of pH on Protein Structure

**Figure 1:**
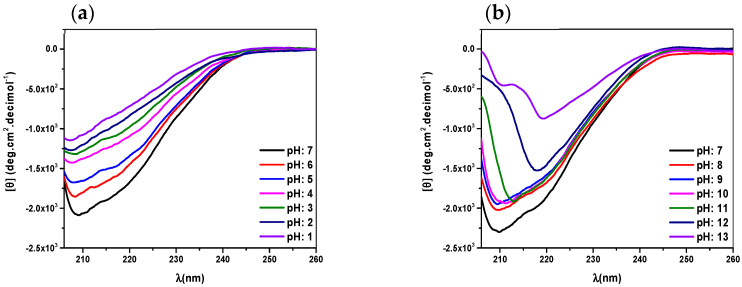
Circular dichroism spectra of recombinant human lactoferrin (0.02 mg/mL) in PBS at 30 °C, at (**a**) pH values 1 to 7 and (**b**) pH values 7 to 13.

The structural conformation changes of the protein were evaluated by measuring the circular dichroism spectra modifying the pH. Figure 1a shows the CD spectra of recombinant human lactoferrin at pH 7.0 where the two absolute minima, at 208 nm and 220 nm, suggest a predominantly α-helix structure. When the pH is increased, these two signals present a decrease in MRE values associated with a denaturalization process (see Figure 1b).

When pH is decreased, the CD spectra of recombinant human lactoferrin show a diminution in all MRE values, associated with a decrease in the amount of soluble protein, due to precipitation of the sample. This was confirmed by a simple visual inspection into the optical cell in each experiment. The percentage of α-helix values were calculated using the CD observed at 208 nm, according to Equations (1) and (2), and are presented in Table 1.

(1) $$MRE=\frac{\left.CD\;Observed\;at\;208\;\mathrm{nm}(mdeg\right)}{C_{p}nl\times 10}$$

(2) $$\alpha -helix\left(\%\right)=\frac{-{MRE}_{208}-4000}{33,000-4000}\times 100$$

where *MRE* corresponds to mean residual ellipticity, *Cp* is the molar concentration of the protein, *n* is the number of amino acid residues and *l* is the optical path length. From the values presented in Table 1. It can be inferred that the protein stability is optimal at neutral pH.

**Figure 2:**
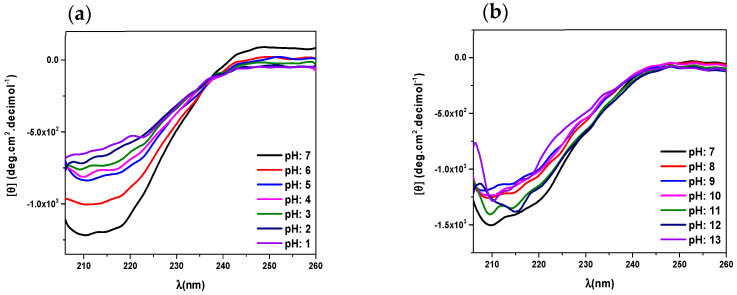
Circular dichroism spectra of native human lactoferrin (0.02 mg/mL) in PBS at 30 °C, at (**a**) pH values 1 to 7 and (**b**) pH values 7 to 13.

The CD spectra of native human lactoferrin at pH 7, in the same conditions of recombinant human lactoferrin (see Figure 2a), also present two minima near to 208 nm and 220 nm, indicating a low percentage of α-helix in comparison with recombinant human lactoferrin. For pH values higher than 7, a diminution in all spectral values is observed, due to a decrease in the amount of soluble protein, associated with its precipitation; this is the same behavior as that observed in recombinant human lactoferrin (see Figure 2).

There are few studies published regarding conformational changes evaluated via circular dichroism; however, we can cite a study by Sreedhara et al. who, in 2010, evaluated the effect of pH on the far-UV circular dichroism spectra of caprine lactoferrin (cLF) and bovine lactoferrin (bLF) at pH 2.0, pH 5.0 and pH 8.0, reporting that the secondary structures of bLF in the pH range of 5–8.0 are constant, with minor changes; a similar pattern was observed in the case of cLF [27]. 

Differences have been found between proteins from different species, even in native lactoferrin. It is not surprising that differences are found between a recombinant protein and a native one, even if it is the same protein. By means of the use of the software Chimera and the corresponding PDF file [28] a 41% of α-helix in solid state was calculated. The difference with the obtained value of 28% of α-helix, can be attributed be due to solvation or ionic interactions or hydrogen bonds when going from solid state to solution.

#### 2.2.2. Effect of Temperature and Thermodynamic Parameters

**Figure 3:**
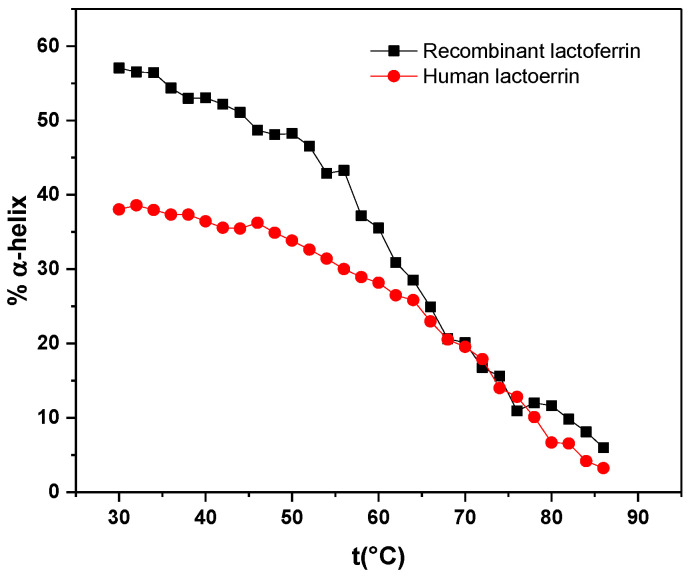
Percentage α-helix vs. temperature of recombinant and native human lactoferrin (0.02 mg/mL) in PBS at 30 to 86 °C.

The CD spectra of both samples, recombinant human lactoferrin and native human lactoferrin, were recorded at different temperature values. According to Equations (1) and (2), the percentage of α-helix values were calculated at each temperature (see Figure 3). A typical protein denaturation process is observed for the two proteins, where there is a decrease in the percentage of α-helix. This result indicates that it is possible to measure thermodynamic parameters for this process. 

**Figure 4:**
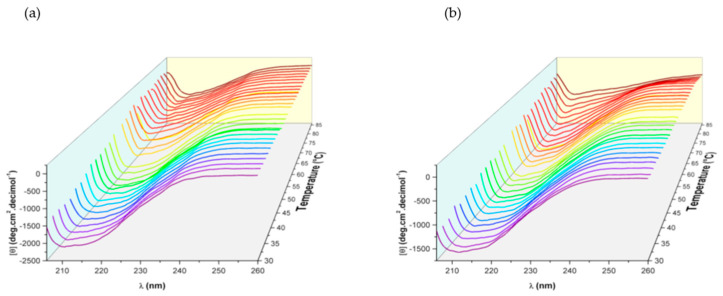
Three-dimensional plots for thermal denaturalization of recombinant human lactoferrin 0.02 mg/mL (**a**) and native human lactoferrin (**b**) (0.03 mg/mL) in PBS at 30 to 86 °C, pH 7.

Figure 4 shows a 3D graph, where MRE, temperature and wavenumber are plotted. Considering one plot at a fixed wavenumber, the fraction of unfolded protein *F*(*u*) can be estimated using the Equation (3),

(3) $$f(u)=\frac{Y_{0}-(y_{N}+m_{N}T)}{\left(y_{u}+m_{u}T\right)-(y_{N}+m_{N}T)}$$

In this equation, *Y*_0_ corresponds to the ellipticity value, and *Y_N_* and *m_N_* are the base line and the slope of the protein before the denaturation temperature *Tm*, obtained from the ellipticity values. In the same way *Y_U_* and *m_U_* are the base line and the slope of the protein after the denaturation temperature Tm, respectively, *T* is the temperature in Kelvin and R = 8.314 JK mol^−1^. With this value, an equilibrium cotan at each temperature can be calculated, see Equation (4).

(4) $$K_{T}=\frac{F(u)}{1-F(u)}$$

By means of the linear adjustment of ln(*K*) = *f*(1/*T*), the following thermodynamic parameters were obtained: *Tm* (transition temperature), ΔH and ΔS. For recombinant human lactoferrin, the obtained values are ΔH = 318.8 kJ/mol, ΔS = 0.94 kJ/molK and *Tm* = 318 K with χ = 0.0017, corresponding to a non-spontaneous system. In the case of native human lactoferrin, the obtained values are ΔH = 103.3 kJ/mol, ΔS = 0.308 kJ/molK and *Tm* = 334 K, with χ = 0.0035. The difference in thermodynamic values indicates more disorganized system (↑ΔS) in recombinant human lactoferrin associated with a low percentage of α-helix, and the break in different bonds within the structure (↑ΔH) indicates non-similar molecular arrangement in both proteins. Hence, either thermodynamic parameters or simple denaturation temperature (*Tm*) can be a criterion before using recombinant human proteins for the preparation of specialized recombinant proteins. 

The changes in the CD response and in the structure for recombinant human lactoferrin and native human lactoferrin can be associated with that in the production of the recombinant protein in batch. In YPD selective culture medium, the apo-form could have been favored and could explain the differences in the thermodynamic parameters with the native protein. This is in agreement with the studies reported by Sreedhara et al., where the thermal denaturation (Tm) data of apo and holo forms of LF indicated that the holo LFs of both bovine and caprine species are different, being more stable than the respective apo form in the pH range 2.0–7.0. Furthermore, the conformation of apo and holo LFs from both caprine and bovine species showed an increased unfolded structure with reduced pH values. At pH 2.0, a higher content of aperiodic structures with an overall loss of a-helices was observed in the case of the apo and holo forms of bLF. Apo cLF and holo cLF showed 7–8% α-helix at low pH [27].

In apo-Lf, the N-lobe has an open conformation (an angle of 53° between the N1 and N2 subdomains), while the C-lobe has a closed conformation (subdomains C1 and C2 move closer). This conformation makes it more resistant to heat denaturation and proteolysis. The closed conformation of holo-Lf is lost when the structure is destabilized, and this can occur, for example, when Lf binds to a bacterial or other receptor (the same happens with Tf), when it is at a very acidic pH (less than 4.0) or when there is protonation of the carbonate ion (the closed structure is destabilized, and the Fe is released). The difference between apo-Lf and holo-Lf in the conformational structure is then simply due to movements in the domains of the protein, and the shape of apo-Lf offers interaction with a greater number of molecules [29,30].

## 3. Materials and Methods

### 3.1. Strains and Growth Condition

The activation of competent *E. coli* Top 10 cells was carried out in 100 mL of LB medium (Luria–Bertani, Sigma-Aldrich, St. Louis, MO, USA) supplemented with 20 mmol/L of MgCl_2_ solution, incubated at 37 °C for 18 h. *Komagaetella phaffii* X-33 yeast was reactivated in 15 mL YPD liquid medium (Yeast Extract Peptone Dextrose, Sigma-Aldrich) for 24 h/30 °C with shaking at 150 RPM.

### 3.2. Yeast Transformation and Molecular Biology

The *E. coli* Top 10 competent cells for plasmid propagation were collected through centrifugation at 8000 RPM at 4 °C for 10 min, the supernatant was discarded and the bacterial biomass was resuspended in 20 mL of a 50 mmol/L CaCl_2_ solution previously placed on ice. The suspension was placed again in the ice bath for 30 min, and the cells were collected again through centrifugation at 8000 RPM for 10 min. After removing the supernatant, the cell pellet was resuspended in a mixture of a 50 mmol/L CaCl_2_ solution with 20% glycerol. Finally, 200 μL aliquots were stored at −70 °C. A total of 20 ng of plasmid DNA was added to the vial and mixed via inversion. It was incubated on ice for 50 min, and then, placed in a water bath at 42 °C for 30 s without shaking. The vial was removed from the water bath and immediately placed on ice for 2 min. A total of 500 μL of S.O.C medium (2% tryptone, 0.5% yeast extract, 10 mM NaCl, 2.5 mM KCl, 10 mM MgCl_2_, 10 mM MgSO_4_, 20 mM glucose) was added, previously conditioned at 25 °C, and the vial was incubated at 37 °C for 1 h with horizontal shaking at 150 RPM. Two hundred microliters of the incubated (transformed) cells was spread on low-salt LB agar plates supplemented with 50 μg/mL Zeocin™ (Thermo Fisher Scientific, Waltham, MA, USA) and incubated at 37 °C overnight. Colonies were selected and analyzed by endpoint PCR to verify correct insertion of the plasmid.

The recombinant plasmid pGZaLM used as the expression vector was synthesized in a commercial laboratory (Invitrogen, Waltham, MA, USA) and was the product for cloning the plasmid pGAPZαA (Invitrogen, Waltham, MA, USA) and the gene with optimized nucleotide sequence. The optimized nucleotide sequence encoding the recombinant breast milk (BM) protein was synthesized in vitro using the BM protein mRNA sequence published in GenBank with the reference LACTOFERRIN|Gen LTF, REFSEQ: AY165046.1.

The plasmid pGZaLM is composed of an optimized coding nucleotide sequence fused “in frame” to a GAP promoter region (pGAP) of the plasmid, which allows for the constitutive and stable expression of the recombinant protein in the yeast *K. phaffii*. The optimized coding nucleotide sequence is flanked “in frame” by a coding region for secretion factor α, which allows the protein to be secreted into the yeast culture medium. In addition, the plasmid contains a region encoding a polyhistidine motif that allows for specific purification of the recombinant protein. Likewise, this plasmid contains a transcription termination region (AOX1) that increases the stability of the recombinant protein. On the other hand, the plasmid used in the present method contains a gene for resistance to the antibiotic Zeocin™. With its specific regulatory regions for the prokaryotic host and the eukaryotic host, this gene allows for the selection of only cells that have been transformed with the plasmid. Finally, the plasmid contains an origin of replication region, which allows it to be recognized and self-replicate in the prokaryotic host.

The transformation procedure began with the digestion of the plasmid DNA. A total of 10 μg of plasmid was taken and digested with the Bsp HI enzyme, which cuts once in the GAP promoter region (356 bp) to linearize the vector, following the protocol of digestion described by the manufacturer. Complete digestion of the plasmid was verified via electrophoresis in a 1% *w*/*v* agarose gel stained with SYBR Gold™ (Invitrogen, Waltham, MA, USA). Because the digested plasmids must be free of salts to be able to perform electroporation, they were cleaned through precipitation of the plasmid DNA with 0.7 volumes of isopropanol and washing with 70% ethanol. Resuspension was carried out in 10 μL of sterile deionized water and kept at 4 °C until electroporation.

For the transformation of the yeast, the physical method of electroporation using exponential decay pulses was used, through which high transformation efficiencies can be achieved (on average, 10^4^ transformants/μg of plasmid DNA). In this method, cells are exposed to a high-voltage electric field, which generates temporary rearrangement of the cell membrane. In this way, cells are permeable, enabling them to take up solutes from the surrounding environment, such as plasmid DNA and other smaller molecules [31]. A total of 20 mL of the culture was taken and centrifuged for 5 min at 1500 RPM. The resulting supernatant was discarded, and the pellet was washed with sterile distilled water at 4 °C. A second centrifugation of the washed cells was performed for 10 min at 1500 RPM. The transformation solution was poured into a 0.2 cm cuvette (Bio-Rad Laboratories, Hercules, CA, USA) and the cells were resuspended in 5 mL of 1 M sorbitol. These electrocompetent cells were maintained at 4 °C and electroporation proceeded. In 0.2 cm electroporation cells previously cooled on ice, 80 μL of electrocompetent *Komagaetella phaffii* cells were mixed with 5 μg of linearized plasmid DNA corresponding to the protein of interest. The cells with the mixture were incubated for 5 min on ice prior to placing them in the Gene Pulser Xcell electroporator (Bio-Rad Laboratories) under the following conditions: 25 μF capacitance, 200 Ohm resistance and 2.0 kV voltage. Immediately after electroporation, 1 mL of cold 1 M sorbitol was added to the cells, and the contents were transferred to sterile 1.5 mL tubes.

### 3.3. Protein Expression 

The expression system was composed of the yeast *K. phaffii* X-33 (Invitrogen, Waltham, MA, USA) as the eukaryotic host, as well as the bacteria *E. coli* Top 10 (One Shot™) (Invitrogen, Waltham, MA, USA) as the prokaryotic host, and the recombinant plasmid pGZαLM (Tib Mol Biol GmbH, Eresburgstrasse, Germany). 

### 3.4. Obtaining Purified hLF Recombinant Protein

The yeast strain *Komagaetella phaffii* X-33 transformed with the plasmid previously characterized for the expression of hLF was used, and its production was induced in the YPD selective culture medium (Sigma-Aldrich, St. Louis, MO, USA), incubating at 30 °C for 48 h with stirring at 150 RPM and with aeration. Recombinant proteins were purified from the culture medium using a Probond^TM^ affinity chromatography system (Thermo Fisher Scientific, Waltham, MA, USA), which uses a polymeric matrix activated with iminodiacetic acid and nickel (nickel is a di- and trivalent metal that allows us to selectively separate recombinant proteins tagged with polyhistidine residues, His Tag). Simple purification of secreted recombinant proteins is possible due to the relatively low levels of native secreted proteins.

### 3.5. Analysis of the Molecular Mass of the Recombinant hLF Protein Obtained 

This analysis was carried out via vertical polyacrylamide gel electrophoresis under denaturing and reducing conditions (SDS-PAGE) using a Mini-protean II system (Bio-Rad, South Granville, NSW, Australia). Each sample was mixed with an equal volume of 2X Laemmli loading buffer (100 mM Tris–HCl, pH 6.8, 4% SDS, 0.2% bromophenol blue, and 20% glycerol) (Bio-Rad, Hercules, CA, USA). Samples were loaded onto a 4–20% gradient TGX™ polyacrylamide gel (Bio-Rad, Hercules, CA, USA) along with a Precision Plus™ Protein molecular mass marker (Bio-Rad, Hercules, CA, USA). Proteins were visualized with QC Colloidal Coomassie Stain (Bio-Rad, Hercules, CA, USA). Molecular mass calculation was performed based on the molecular mass marker using the ImageLab 4.1 point-to-point method [32].

### 3.6. Circular Dichroism

Circular dichroism measurements were performed using a MOS-500 Bio-Logic spectrophotometer. Protein solutions were prepared in phosphate buffer (PBS), and then, 2 mL was placed in a quartz cell with a 1 cm optical path length. During the measurements, the temperature was controlled with a peltier. Spectra were obtained in the range of 206 nm to 260 nm with a slit of 0.5, step of 0.25 and tadq of 0.5 s. For the experiments in which the pH was varied, the lactoferrin solution was adjusted with NaOH or HCl as appropriate, at 30 °C. In the Biokine program of the spectrophotometer, a temperature ramp was programmed from 30 °C to 86 °C, acquiring circular dichroism spectra every 2 °C.

## 4. Conclusions

Insertion of the recombinant plasmid pGZαLM by means of the CaCl_2_/heat shock method into the *E. coli* strain Top 10 (One Shot™) proved to be suitable.

The spectra obtained through circular dichroism show that at alkaline pH, the secondary structure of the native lactoferrin, as well as the recombinant, changes to an unfolded structure, and at acidic pH, both proteins precipitate. 

By means of the CD response at different pH levels and through the determination of thermodynamic parameters, it was possible to propose that in the production of recombinant human lactoferrin, the apo-form could have been favored in comparison with native human lactoferrin. 

The differences found between those two proteins despite coming from the same gene may be attributed to its different origin: one is the human native lactoferrin while the other is a recombinant lactoferrin obtained from yeast *Komagataella phaffi* (formerly *Pichia pastoris*), a widely used eukaryotic system for the production of recombinant proteins, considered at the beginning of this work as a good candidate for the production of lactoferrin. It has been reported that yeasts may have some peculiarities at the interpretation of some genetic signals production while processing recombinant proteins, as is confirmed in this work.

To determine whether recombinant lactoferrin and native lactoferrin are comparable in function to be use in food products, some safety studies must be carried out. However, CD can be used as a first inspection tool in the production of recombinant proteins.

## Figures and Tables

**Table 1:** Values of percentage α-helix of recombinant and native human lactoferrin at 30 °C varying the pH (7 to 13) of the medium (PBS).

	Percentage of α-Helix	
pH	Recombinant Human Lactoferrin	Native Human Lactoferrin
7	59.8	36
8	55.9	24.6
9	52.5	21.9
10	49.7	23.7
11	22.6	27.9
12	0.78	19.5
13	n.o.	11.7

## Data Availability

Data are contained within the article and Supplementary Materials.

## Supplementary Materials

The following supporting information can be downloaded at: https://www.mdpi.com/article/10.3390/molecules29020491/s1. Figure S1. Electrophoretic analysis of the amplification of the recombinant hLF gene from *K. phaffii* genomic DNA. Line 1. MPM. Line 5–7. LF strains. Figure S2. Quantification of recombinant human Lactoferrin in crude extract from the *K. phaffii* culture, and concentrated in AMICON^TM^ Ultracel 10K tubes. Figure S3. SDS-PAGE analysis of the concentrated extracts in AMICON^TM^ Ultracel 10K tube of the culture medium of the transgenic strains of *K. phaffii* transformed with the hLF gene.
