# Supplementary material for: Study of pH and Thermodynamic Parameters via Circular Dichroism Spectroscopy of a Recombinant Human Lactoferrin
Source: Molecules. 2024 Jan 19;29(2):491. doi: 10.3390/molecules29020491 (PMC10818261; doi:10.3390/molecules29020491)
Supplement: Supplementary file 1 [file molecules-29-00491-s001.zip › molecules-2761218-supplementary.pdf]

Supplementary information

Study of pH and thermodynamic parameters by circular dichroism spectroscopy of a recombinant human lactoferrin

Beatriz L. Álvarez-Mayorga [1,*], Sergio Romero-Gómez [1], Jorge L. Rosado [2,*], Janet Ocampo-Hernández [3], J. Gómez-Guzmán [3] and Luis Ortiz-Frade [3]

[1] Facultad de Química, Universidad Autónoma de Querétaro, Querétaro 76010, Mexico;
sergio.romero@uaq.edu.mx
[2] Departamento de Nutrición Humana, Facultad de Ciencias Naturales, Universidad Autónoma de
Querétaro, Querétaro 76230, Mexico
[3] Departamento de Electroquímica, Centro de Investigación y Desarrollo Tecnológico en Electroquímica S.C. Parque Tecnológico Querétaro, Sanfandila, Querétaro 76703, Mexico; jocampo@cideteq.mx (J.O.-H.); jesusgomezguzman@hotmail.com (J.G.-G.); lortiz@cideteq.mx (L.O.-F.)
* Correspondence: beatriz.alvarez@uaq.edu.mx (B.L.Á.-M.); jrglrosado@gmail.com (J.L.R.)

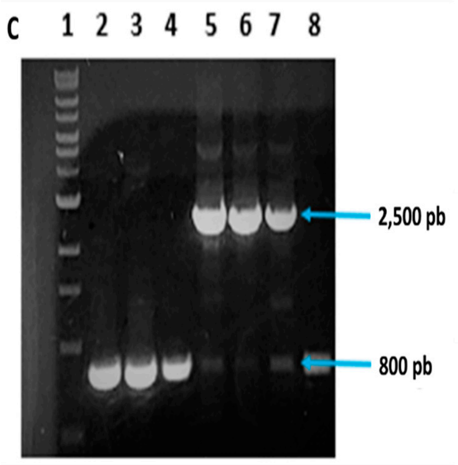

Figure S1. Electrophoretic analysis of the amplification of the recombinant hLF gene from *K. phaffii* genomic DNA. Line 1. MPM. Line 5-7. LF strains.

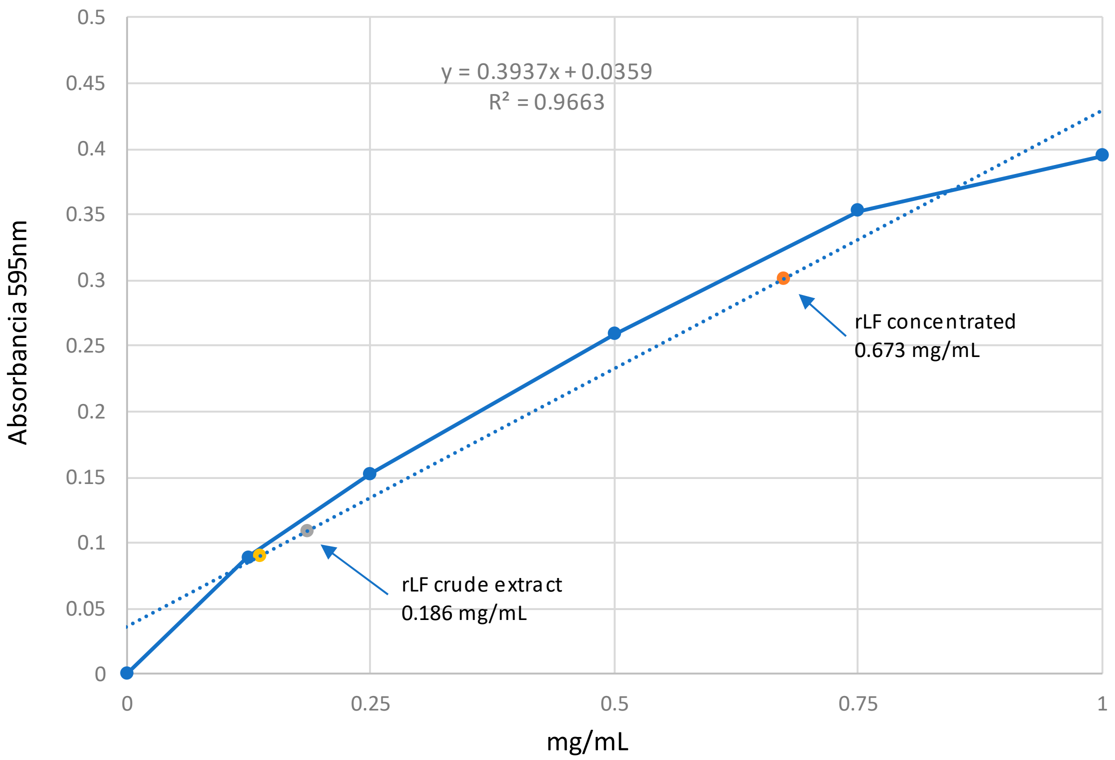

Figure S2. Quantification of recombinant human Lactoferrin in crude extract from the K. phaffii culture, and concentrated in AMICONTM Ultracel 10K tubes

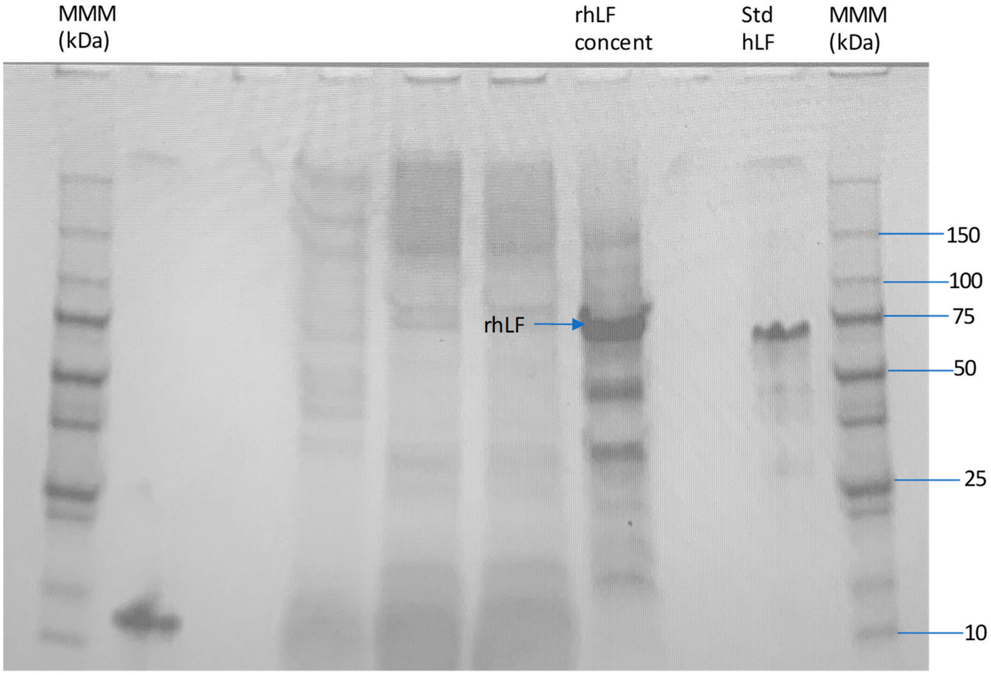

Figure S3. SDS-PAGE analysis of the concentrated extracts in AMICON™ Ultracel 10K tube of the culture medium of the transgenic strains of *K. phaffii* transformed with the hLF gene.
